# The Effect of Sensory Uncertainty Due to Amblyopia (Lazy Eye) on the Planning and Execution of Visually-Guided 3D Reaching Movements

**DOI:** 10.1371/journal.pone.0031075

**Published:** 2012-02-17

**Authors:** Ewa Niechwiej-Szwedo, Herbert C. Goltz, Manokaraananthan Chandrakumar, Agnes M. F. Wong

**Affiliations:** 1 Department of Ophthalmology and Vision Sciences, The Hospital for Sick Children, Toronto, Canada; 2 University of Toronto, Toronto, Canada; Federal University of Rio de Janeiro, Brazil

## Abstract

**Background:**

Impairment of spatiotemporal visual processing in amblyopia has been studied extensively, but its effects on visuomotor tasks have rarely been examined. Here, we investigate how visual deficits in amblyopia affect motor planning and online control of visually-guided, unconstrained reaching movements.

**Methods:**

Thirteen patients with mild amblyopia, 13 with severe amblyopia and 13 visually-normal participants were recruited. Participants reached and touched a visual target during binocular and monocular viewing. Motor planning was assessed by examining spatial variability of the trajectory at 50–100 ms after movement onset. Online control was assessed by examining the endpoint variability and by calculating the coefficient of determination (R^2^) which correlates the spatial position of the limb during the movement to endpoint position.

**Results:**

Patients with amblyopia had reduced precision of the motor plan in all viewing conditions as evidenced by increased variability of the reach early in the trajectory. Endpoint precision was comparable between patients with mild amblyopia and control participants. Patients with severe amblyopia had reduced endpoint precision along azimuth and elevation during amblyopic eye viewing only, and along the depth axis in all viewing conditions. In addition, they had significantly higher R^2^ values at 70% of movement time along the elevation and depth axes during amblyopic eye viewing.

**Conclusion:**

Sensory uncertainty due to amblyopia leads to reduced precision of the motor plan. The ability to implement online corrections depends on the severity of the visual deficit, viewing condition, and the axis of the reaching movement. Patients with mild amblyopia used online control effectively to compensate for the reduced precision of the motor plan. In contrast, patients with severe amblyopia were not able to use online control as effectively to amend the limb trajectory especially along the depth axis, which could be due to their abnormal stereopsis.

## Introduction

Variability is an inherent feature of human movements [1]. It can be observed in the kinematics, kinetics, and patterns of muscle activation, even when the task is simple or has been practiced extensively [2]. There are three main sources of variability in goal-directed movements: uncertainty in target localization during sensory coding, noise associated with transformation of sensory inputs into a motor command during the motor planning stage, and noise associated with conversion of motor commands into muscle action during the movement execution stage [3], [4].

During sensory coding, the precision of target localization is determined by the properties of the receptors. In the visual system, the size and density of the photoreceptors varies across the retina such that spatial resolution is best at the fovea and worse in the periphery [5], [6]. Target localization is also dependent upon the visual context. For example, pointing accuracy improves when the target is presented in a structured background as compared to pointing in the dark [7], [8], and pointing precision is better for targets located in the lower visual field [9], [10], [11]. In addition, the precision of localizing a target in the extrapersonal space differs among sensory modalities: visual localization is more precise along the azimuth than in depth, whereas proprioceptive localization is more precise in depth than along the azimuth. As the target distance increases, both visual and proprioceptive localization precision is reduced [12].

During the motor planning stage, sensorimotor transformation occurs so that target location is transformed from a retinocentric representation to a gaze-centered or body-centred frame of reference [13], [14], [15]. The motor plan is then computed based on target location and the initial position of the arm estimated from proprioceptive and visual information. Movement precision increases when the initial hand position is seen, indicating that visual and proprioceptive information are combined to give a more reliable estimate of the initial static hand position [16], [17]. When movements are initiated after a memory delay, motor planning noise increases leading to decreased movement precision [18], [19]. It has been shown that the variability in neural activity in the premotor and motor cortex prior to movement initiation can explain approximately 50% of the variability in peak velocity during a subsequent reaching movement [20]. Taken together, these studies suggest that central planning processes contribute significantly to movement variability.

During the movement execution stage, motor commands are relayed to the muscles and converted into mechanical forces to produce the desired movement. van Beers et al (2004) showed that execution noise is dependent on the amplitude as well as the duration of the motor output. As a consequence, the variability in movement endpoints, and hence the success of reaching the target, depends on the direction of the chosen movement trajectory [21]. In addition, the variability in the force output is proportional to the average force that is produced by the muscles [22], consistent with the minimum-variance theory proposed by Harris and Wolpert (1998). According to this theory, motor commands are corrupted by noise, and the level of noise scales with the magnitude of the command (i.e., signal-dependent noise). Because of the effects of execution noise on the actual movement trajectory, the goal of movement control is to plan the trajectory such that the expected likelihood of missing the target is minimized (i.e., minimize the end-point variance), which also explains the speed-accuracy trade-off known as Fitts' law (Fitts 1954).

Uncertainty arising from sensory coding, planning, and execution of movements interact in a complex manner. Moreover, uncertainty arising from sensorimotor processing will affect both the planning and the online control of movements [23]. The effects of sensory uncertainty on the planning and execution of reaching movements can be investigated in a unique disease model—amblyopia. Amblyopia is a neural developmental disorder characterized by reduced visual acuity due to inadequate stimulation of the eye(s) during early childhood and cannot be corrected by optical means. It is usually defined as a visual acuity of 20/30 or worse without any apparent structural abnormality in the affected eye. Amblyopia is associated most commonly with early childhood strabismus (eye misalignment), anisometropia (difference in refractive errors between the eyes), or both (i.e., mixed). Patients with amblyopia have a myriad of visual and perceptual deficits, including reduced visual acuity and contrast sensitivity [24], deficits in global form detection [25], spatial distortions and temporal instability [26], [27], spatial and temporal crowding [28], abnormal global motion detection [29], [30] and deficits in motion-defined form extraction [31]. Importantly, these deficits are not only present during amblyopic eye viewing; they are also evident to a lesser extent, during binocular and fellow eye viewing [24], [32], [33]. It is generally agreed that the earliest functional and anatomical abnormalities that contribute significantly to the behavioural losses in amblyopia occur in cortical area V1 [34], [35], [36] (see also the role of LGN in the feedback pathway [37], [38], [39]). These abnormalities are then amplified downstream in the extrastriate cortex and specialized cortical areas [27], [28], [40], [41], [42].

Perceptual deficits associated with amblyopia have been studied extensively (for review see [43], [44]); however, the effects of the visual impairments on motor behaviours have not received similar attention. Several recent studies have addressed this gap in the literature by examining the effect of reduced acuity and stereoacuity on eye-hand coordination skills, including block-building, bead-threading, ball-catching [45] or by using clinical tests to asses visuomotor skills [46], [47], [48]. These studies found that patients had impaired performance during motor tasks that emphasize both speed and accuracy. Two other studies have examined the kinematics of reaching and grasping movements using 3D motion tracking. In the first study, Grant et al. [49] examined grasping in adults with different types of amblyopia. They found that patients had longer movement times and made more errors in the grasping phase during amblyopic eye viewing. However, movements were comparable to those made by control subjects when patients viewed with the better (fellow) eye or binocularly. In the second study, Suttle et al. [50] examined reaching and grasping movements in children (4–8 years old) with amblyopia and reported that movements were slower and exhibited more errors under all viewing conditions. These previous studies have shown that amblyopia impairs motor performance; however, it is not known whether or to what extent the deficit affects motor planning and online control, which is the focus of the current paper.

The goal of this study was to examine the effects of amblyopia on motor planning and online control during visually guided reach-to-touch movements. Our approach was to assess motor planning by examining the variability of the reach trajectory (i.e., the magnitude and angle of the three-dimensional reach vector) early in the movement (i.e., 50 and 100 ms after the onset of movement). We also assessed online corrections by examining endpoint variability and by performing a correlation analysis (coefficient of determination, R^2^; see Methods for a more detailed description) relating the position of the finger at different points in the trajectory with its position at the end of movement [51], [52]. Both variability and correlation analyses are based on the assumption that performance is limited by the presence of sensorimotor noise which leads to variability in the motor output [3], [51]. Thus, examining spatial variability of limb position during and at the end of the movement can illuminate how effectively feedback was used to correct for errors. Specifically, if the movement was pre-programmed and executed without the benefit of feedback/online control, errors early in the trajectory would be amplified as the movement unfolds. Using this approach, we found that the sensory uncertainty due to amblyopia led to reduced precision of the motor plan in all viewing conditions as evidenced by increased variability of the reach vector angle early in the trajectory. The ability to implement online corrections, however, was dependent on the severity of the visual deficit, viewing condition, and the axis of the reaching movement.

## Methods

### Participants

The study was approved by the Hospital for Sick Children Research Ethics Board and all protocols adhered to the guidelines of the Declaration of Helsinki. Written informed consent was obtained from each participant.

Twenty-six patients with amblyopia were recruited. Patients were classified into two groups based on their acuity deficits. Thirteen patients (10 females, age 25.9±9.3 years) had acuity 20/60 or better in the amblyopic eye, and they were assigned to the mild amblyopia group. The other 13 patients (6 females, age 30.4±11.6 years) had acuity 20/100 or worse in the amblyopic eye, and were assigned to the severe amblyopia group. Nineteen patients had anisometropic amblyopia, 4 had strabismic amblyopia, and 3 had mixed amblyopia. The clinical details of all patients are shown in Table 1. All participants underwent a complete orthoptic assessment by an unmasked certified orthoptist. The assessment included visual acuity testing using the Snellen chart (recorded as the last row in which a participant could correctly read all letters), measurement of eye alignment using the prism cover test, measurement of refractive errors, and stereoacuity testing using the Titmus test. The fusion ability of patients who lacked stereopsis (negative Titmus test) was tested using the Worth 4 dot test and the Bagolini test. Thirteen visually normal participants (6 females, age 31.3±11.1 years) served as control subjects. They had normal or corrected-to-normal visual acuity of 20/20 or better in each eye, and stereoacuity ≤40 seconds of arc. Exclusion criteria were any ocular cause for reduced visual acuity, prior intraocular surgery, or any neurologic disease.

**Table 1:** Clinical characteristics of patients with amblyopia.

Patient	Age (years)	Type of Amblyopia	Visual acuity		Refractive Error		Stereoacuity (arc sec)
			Left eye	Right eye	Left eye	Right eye	
1	14	aniso	20/15	20/50	+2.00	+3.25+1.25×90	50
2	18	aniso	20/40	20/15	+2.00+0.25×130	plano	60
3	19	aniso	20/40	20/20	−3.50+2.50×102	−3.50+1.50×90	200
4	20	aniso	20/50	20/20	+1.50	plano	120
5	25	aniso	20/50	20/20	−3.00+2.50×80	−1.50+1.50×80	120
6	25	aniso	20/15	20/40	plano	+1.00+0.25×22	400
7	28	aniso	20/15	20/50	+0.25	+2.50+0.75×50	3000
8	35	aniso	20/60	20/15	−0.75	−4.25	3000
9	36	aniso	20/40	20/15	+1.50+1.00×15	−1.50	200
10	46	aniso	20/15	20/40	plano	+2.75+2.25×60	400
11	17	aniso	20/40	20/20	−1.00+1.00×92	pl +0.25×94	80
12	21	aniso	20/15	20/30	plano	+1.50	3000
13	33	aniso	20/30	20/15	+2.00	−0.75	140
14	20	aniso	20/20	5/400	−3.00+0.75×15	−2.00	negative
15	20	mixed	20/15	4/400	−5.00+0.50×90	−16.00	negative
16	23	mixed	20/15	20/200	Not available	Not available	negative
17	36	aniso	20/400	20/15	−12.00	−5.25	negative
18	56	aniso	20/20	20/100	−2.25	+4.00	negative
19	16	aniso	20/15	20/200	−2.75	none	negative
20	25	strab	20/200	20/20	−0.25	−0.75+0.50×90	negative
21	26	aniso	20/15	20/100	plano	+2.00	3000
22	28	aniso	20/400	20/20	+6.50	pl +0.50×90	negative
23	35	strab	20/200	20/20	plano	−0.75	negative
24	37	strab	20/200	20/20	plano	plano	3000
25	48	strab	20/200	20/15	plano	plano	negative
26	25	mixed	20/200	20/20	1.50×130	plano	negative

aniso – ansiometropic amblyopia.

strab – strabismic amblyopia.

mixed – mixed amblyopia.

### Apparatus

Reach-to-touch movements of the right upper limb were recorded using an Optotrak Certus 3020 system (Northern Digital Inc, Canada), an infrared illumination-based motion capture system. This system is non-invasive and allows for precise three-dimensional (3D) motion tracking of the limb (spatial accuracy 0.1 mm, resolution 0.01 mm, sampling frequency 200 Hz). The coordinate system was defined as follows: x-axis (azimuth), y-axis (elevation), and z-axis (depth). The system was calibrated prior to starting the experiment by using a 4-marker digitizing probe to define the coordinate frame for the reaching movement. Two infrared markers (4 mm diameter) were affixed to the index fingertip and wrist joint of the participant's right (dominant) hand. A 15 mm diameter force sensitive resistor (FSR, Tekscan, Boston, MI), was placed on the table at participant's midline 28 cm from the computer screen and 17 cm in front of the participant. The FSR was used to trigger the initiation of each trial and to control when the visual target was switched off during a trial.

### Experimental Conditions and Procedure

The visual stimulus was a white circle (visual angle 0.25°) presented on black background generated by a custom-written Matlab program and presented on a 20 inch CRT computer screen (NEC-Mitsubishi, Diamond Pro 2070SB; resolution 1600×1200 at 85 Hz) located 43 cm from the subject using a ViSaGe visual stimulus generator (Cambridge Research Systems, UK). The distance from the starting position of the index finger to the computer screen was 48 cm in 3D space. Testing was conducted in a dimly lit room. The target was presented at four eccentricities: ±5° (3.8 cm) or ±10° (7.6 cm) from the center, all along the horizontal axis at eye level and in random order.

**Figure 1:**
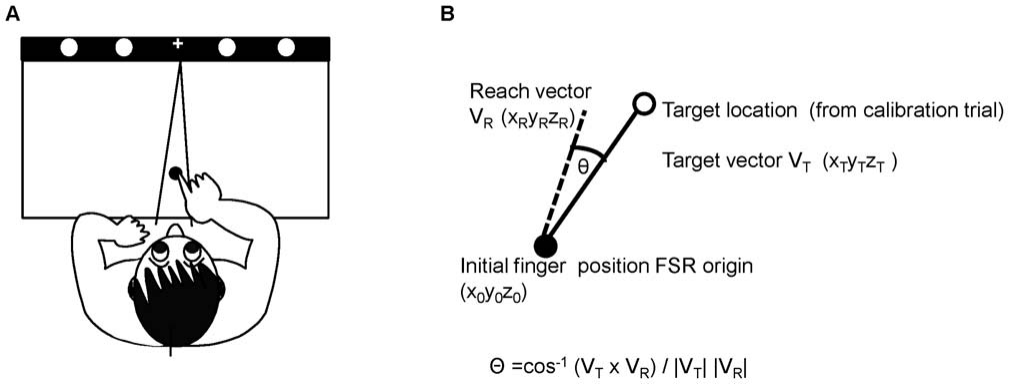
Experimental set up. (A) Participants fixated on a cross displayed on a computer monitor, with their index finger placed on a force sensitive resistor (FSR). The target was a high contrast circle (visual angle 0.25°) shown after a random delay (range 1.5–3 sec) at ±5° or ±10°. (B) The 3D reach vector, defined as a straight line connecting the initial and end position of the finger, was calculated from the finger trajectory data. For each trial, we also computed the angle between the reach vector and the target vector (defined as a straight path connecting the initial position of the finger and target location).

Participants were seated in front of a table with their heads stabilized with a chin rest (Figure 1a). There were three viewing conditions: binocular (BE), monocular amblyopic eye (AE), and monocular fellow eye (FE) viewing, and data were collected in blocks. For control participants, viewing was binocular, monocular left eye and right eye. Participants wore a black patch during monocular viewing. The order of viewing conditions was randomized among the participants. At the start of each trial, the right hand was placed on the table and the index finger was placed on the FSR. Participants fixated on a cross presented at midline. After a variable delay (1.5 to 3 sec), the target was presented in the horizontal plane on the computer screen and participants were instructed to look and touch the target as fast and as accurately as possible. The target was located 28 cm in front (i.e., depth) and 34 cm above (i.e., elevation) the starting position of the hand. For half of the trials, the target remained visible throughout the trial (target on condition). During the remaining 50% of the trials, the target was switched off at the onset of hand movement, i.e., as soon as the finger was lifted off the FSR (target off condition). In these trials, participants were instructed to touch the location where they had seen the target. The target on and off conditions were randomly interleaved.

Participants completed 10 trials in each of the experimental conditions for a total of 240 trials. The inter-trial interval varied among trials and was at least 5 sec. Practice trials were completed prior to starting the experiment in order to familiarize the participants with the experimental procedure. All patients, including those with severe amblyopia, executed spatially and temporally appropriate reaching movements during practice trials, indicating that they were able to detect the target.

### Analysis

Hand position data were filtered using a second-order dual-pass Butterworth filter with a cut-off frequency of 10 Hz. Velocity was obtained using a 2-point differentiation method and acceleration was obtained by differentiation of the velocity signal (2-point differentiation method). A custom-written Matlab program was used to identify the initiation of hand movement which was defined as the time when the velocity of the finger y-coordinate (i.e., elevation axis) exceeded and remained above 30 mm/s. The end of the reaching movement was defined as the time when the finger reached the computer screen and the velocity of the finger z-coordinate (i.e., depth axis) fell and stayed below 30 mm/s. All trials were inspected visually to ensure that the reaching movements were identified correctly by the program.

#### Kinematics

The kinematics of the reaching movement were assessed by calculating the reaction time (defined as the interval between the onset of the visual stimulus and the initiation of reaching) and total movement time (the interval between reach initiation and the end of movement). The 3D reach vector, defined as a straight line connecting the initial and end position of the finger, was calculated from the finger trajectory data. Mean peak acceleration and peak velocity, as well as the time to reach peak acceleration, duration of acceleration phase (defined as the interval from movement onset to peak velocity) and duration of deceleration phase (defined as the interval from peak velocity to end of movement) were also calculated for the reach vector on each trial. Mean kinematic measures were submitted to repeated-measures mixed ANOVA with one between-subjects factor: Group (three levels: control participants, patients with mild and severe amblyopia) and two within-subjects factors: Viewing Condition (three levels: binocular, monocular fellow eye, monocular amblyopic eye; for control participants, binocular, monocular left eye, monocular right eye) and Target Location (four levels: ±5° and ±10°). Preliminary analysis showed that Target Visual Feedback had no significant effect on any outcome measures. Therefore, the results reported herein are pooled for the two visual feedback conditions (on and off).

#### Limb Trajectory Variability

The precision of the motor plan can be inferred from the variability of the trajectory early in movement [52], [53]. In addition, reduced variability at the end of movement indicates that corrections have been implemented during the movement. For example, Khan and colleagues have shown that the variability of limb position increased until the limb reached peak deceleration, and it decreased subsequently in the deceleration phase. Furthermore, a greater reduction in limb position variability at the end of the movement was observed when visual feedback was available throughout the reach, as compared to when visual feedback was absent (Khan et al. 2003).

In this paper, the planning stage of the reaching response was assessed by computing the magnitude and variable error (i.e., within subject standard deviation) of the 3D reach vector at 50 ms and 100 ms after movement onset. For each trial, we also computed the angle between the reach vector (a straight line connecting the initial and end position of the finger) and the target vector (a straight path connecting the initial position of the finger and target location) (Figure 1b). The angle between the two vectors provides an indication of how much the actual trajectory deviated from the straight line path to the target. The variability (i.e., within subject standard deviation) of the angle between the reach vector and target vector was also calculated to examine the precision of the initial motor plan.

Reach vector endpoint variability was used to examine how well patients compensated for errors arising from the motor plan or during movement execution. In addition, we also examined the variability at the end of the movement for each of the three axes (azimuth, elevation, and depth) separately. All dependent outcomes were submitted to repeated-measures mixed ANOVA with Group as a between-subjects factor, and Viewing Condition and Target Location as within-subjects factors.

#### Correlation Analysis

In addition to variability analysis, the temporal dynamics of online control can be assessed using a regression analysis developed by Heath [54]. This analysis involves calculating the coefficient of determination (R^2^) relating the spatial position of the limb at different points in the trajectory with its position at the end of the movement [51], [52]. It has been suggested that the magnitude of the R^2^ at 50–75% of the trajectory can be used to infer the presence of trajectory corrections: lower R^2^ values in the latter half of the trajectory combined with good endpoint precision indicate that online control/feedback was used in the deceleration phase of the movement to attenuate the errors in the initial motor plan. In contrast, higher R^2^ values combined with reduced endpoint precision indicate that movements relied more on pre-programming. Previous studies have shown that this correlation analysis provides a sensitive method to assess the role of vision for online control. For example, lower R^2^ values (i.e., higher degree of online control) have been reported when subjects were aware that visual feedback was available throughout the movement [55]. Lower R^2^ values were also observed when visual feedback from the moving limb was available compared to trials when the limb was occluded [54], and when binocular visual information was available as compared to monocular trials [56].

In this paper, R^2^ was calculated at 10% intervals (normalized to total movement time) for each subject and viewing condition, separately for azimuth, elevation and depth axes. R^2^ values obtained at 70% of movement time were transformed to Fisher z-scores and submitted to repeated measures ANOVA with Group as a between-subjects factor and Viewing Condition as a within-subjects factor. All statistical analyses were performed using the SAS 9.2 software package. The significance level was set at p<0.05. Any significant main effects and interactions were analyzed further using post-hoc pair-wise comparison t-tests.

## Results

### Kinematics of the Reach Vector (Table 2)

**Table 2:** Kinematics of the reach vector (mean ± standard deviation).

	Control			Mild amblyopia			Severe amblyopia		
	BE	LE	RE	BE	FE	AE	BE	FE	AE
Reaction time (ms)	305±52	323±51	322±44	352±77	370±96	359±65	396±56	389±52	439±81
Movement time (ms)	514±96	532±106	529±98	629±107	696±176	659±121	650±93	682±155	696±141
Peak acceleration (m/s^2^)	24.26±10.97	23.63±10.55	24.69±10.85	17.60±6.09	15.48±5.49	17.04±4.58	18.45±4.69	19.20±8.89	17.01±4.04
Time to peak acceleration (ms)	54±12	57±11	52±10	53±17	58±22	50±12	55±15	55±15	54±13
Peak velocity (m/s)	1.92±0.49	1.90±. 0.46	1.89±0.43	1.46±0.28	1.39±0.33	1.42±0.26	1.48±0.28	1.48±0.41	1.42±0.24
Time to peak velocity (ms)	163±29	167±30	165±34	187±43	202±47	182±30	185±35	194±44	192±25
Time after peak velocity (ms)	351±74	365±84	364±78	442±84	493±147	476±108	464±84	488±129	504±135
Peak deceleration (m/s^2^)	10.14±4.84	10.32±4.69	9.95±3.75	6.56±2.25	6.31±2.76	6.44±2.17	6.55±2.31	6.90±3.50	6.43±1.83

BE: binocular; LE: left eye; RE: right eye; FE: fellow eye; AE: amblyopic eye.

#### Reaction Time

There was a significant main effect of Group for reaction time (F_(2,36)_ = 8.30, p = 0.001). Post-hoc tests indicated that patients with severe amblyopia had longer reaction times (408±68 ms) in comparison to control participants (317±50 ms) and patients with mild amblyopia (360±81 ms). There was no significant difference between control participants and patients with mild amblyopia. The interaction between Group and Viewing Condition was also significant (F_(4,72)_ = 3.93, p = 0.006). Post-hoc tests showed no significant difference in reaction time among viewing conditions for control participants and for patients with mild amblyopia. In contrast, patients with severe amblyopia had significantly longer reaction time when viewing with the amblyopic eye (439±81 ms), in comparison to binocular (396±56 ms) and fellow eye (389±52 ms) viewing.

#### Movement Time

There was a significant main effect of Group (F_(2,36)_ = 7.16, p = 0.002) and a significant main effect of Viewing Condition (F_(2,36)_ = 4.00, p = 0.023) for movement time. Post-hoc tests indicated that control participants had significantly shorter movement time in all viewing conditions (binocular: 514±96 ms; left monocular: 532±106 ms; right monocular: 529±98 ms) in comparison to patients with mild amblyopia (binocular: 629±107 ms; fellow eye: 696± ms; amblyopic eye: 659±121 ms) and severe amblyopia (binocular: 650±93 ms; fellow eye: 682±155 ms; amblyopic eye: 696±141 ms). Post-hoc tests also showed that movement time was shorter during binocular viewing in comparison to monocular viewing for all participants.

#### Peak Acceleration

There was a significant main effect of Group for mean peak acceleration (F_(2,36)_ = 3.95, p = 0.028). Post-hoc tests indicated that control participants had significantly higher mean peak acceleration (24.20±10.77 m/s^2^), in comparison to patients with mild (16.71±5.48 m/s^2^) and severe (18.22±6.30 m/s^2^) amblyopia. No other significant main effect or interaction was observed.

#### Time to Reach Peak Acceleration

No significant main effect or interaction was observed.

#### Peak Velocity

Main effect of Group was significant for mean peak velocity (F_(2,36)_ = 7.68, p = 0.002). Post-hoc tests indicated that control participants had significantly higher mean peak velocity (1.91±0.46 m/s) in comparison to patients with mild amblyopia (1.42±0.29 m/s) and severe amblyopia (1.46±0.32 m/s).

#### Time to Reach Peak Velocity (i.e., Acceleration Phase)

The main effect of Group for duration of acceleration phase did not reach statistical significance (F_(2,36)_ = 2.92, p = 0.067).

#### Time after Peak Velocity (i.e., Deceleration Phase)

There was a significant main effect of Group (F_(2,36)_ = 6.82, p = 0.003) and a significant main effect of Viewing Condition (F_(2,36)_ = 3.84, p = 0.026). Post-hoc tests indicated that control participants had significantly shorter deceleration phase (binocular: 351±74 ms; left eye: 365±84 ms; right eye: 364±78 ms), in comparison to patients with mild amblyopia (binocular: 442±84 ms; fellow eye: 493±147 ms; amblyopic eye: 476±108 ms) and severe amblyopia (binocular: 464±84 ms; fellow eye: 488±129 ms; amblyopic eye: 504±135 ms). Post-hoc tests indicated that the duration of deceleration phase was significantly shorter during binocular viewing in comparison to monocular viewing for all participants.

### Limb Trajectory Variability

**Figure 2:**
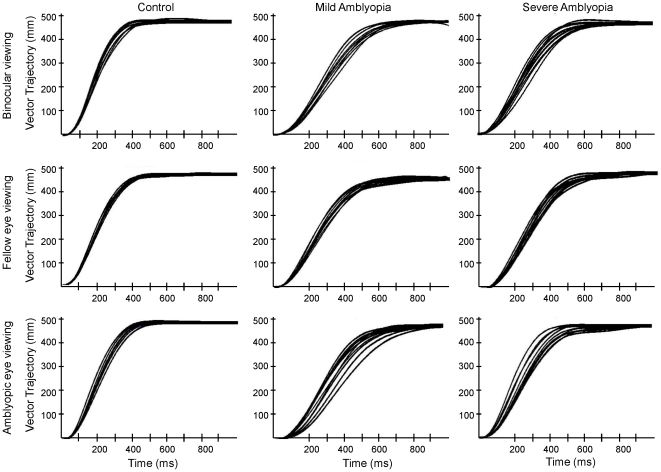
Representative reach trajectory. Typical data showing the reach vector trajectory to the +10° target in a representative control subject (left column), patient with mild amblyopia (middle column), and severe amblyopia (right column) during binocular viewing (top row), fellow eye (right eye in control) viewing (middle row), and amblyopic eye (left eye in control) viewing (bottom row). Patients with mild and severe amblyopia had greater variability in spatial limb position during reaching in comparison to the control subject.

Figure 2 shows the typical reach vector trajectory for reaches to a +10° target during binocular, amblyopic eye and fellow eye viewing. It demonstrates that patients tend to have greater variability in limb trajectory during all viewing conditions in comparison to control participants.

#### Reach vector 50 ms after movement onset (Table 3)

**Table 3:** Kinematics of the reach trajectory 50 ms after movement onset (mean ± standard deviation).

	Control			Mild amblyopia			Severe amblyopia		
	BE	LE	RE	BE	FE	AE	BE	FE	AE
Vector magnitude (mm)	11.95±5.21	11.45±5.11	11.79±4.96	9.68±4.14	9.17±4.61	9.55±3.35	9.09±2.51	9.12±3.51	8.17±1.73
Vector variability (mm)	1.95±0.93	1.96±1.08	2.11±1.32	1.88±1.34	1.56±0.78	2.03±1.91	1.74±1.00	1.85±1.44	1.63±1.17
Angle magnitude (°)	0.64±0.22	0.64±0.20	0.64±0.22	0.50±0.20	0.49±0.21	0.52±0.18	0.49±0.16	0.49±0.18	0.47±0.11
Angle variability (°)	0.11±0.05	0.10±0.04	0.11±0.05	0.13±0.06	0.13±0.06	0.14±0.06	0.14±0.04	0.14±0.06	0.14±0.05

BE: binocular; LE: left eye; RE: right eye; FE: fellow eye; AE: amblyopic eye.

Mean *magnitude* of the reach vector was not significantly different among Groups (F_(2,36)_ = 1.97, p = 0.155). There was also no significant difference in the *variability* of the reach vector magnitude (F_(2,36)_ = 0.84, p = 0.441).

**Figure 3:**
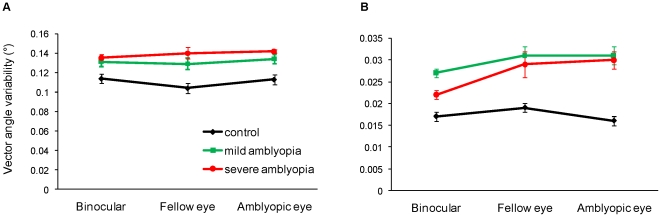
Reach precision during acceleration. Patients with mild and severe amblyopia had significantly reduced precision of the vector angle at 50 ms following movement onset (A), and 100 ms after the onset of movement (B). For control subjects, fellow eye is the right eye and amblyopic eye is the left eye. Error bars = ±1 standard error.

Similarly, *mean angle* between the reach vector and the target vector was not significantly different among Groups (F_(2,36)_ = 1.97, p = 0.154). In contrast, there was a significant main effect of Group for the *variability* (i.e., within subject standard deviation) of the vector angle (F_(2,36)_ = 4.07, p = 0.026; Figure 3a); the variability was significantly lower in control participants (0.11±0.05°), as compared to patients with mild (0.13±0.06°) and severe (0.14±0.05°) amblyopia. No other significant main effect or interaction was observed.

#### Reach vector 100 ms after movement onset (Table 4)

**Table 4:** Kinematics of the reach trajectory 100 ms after movement onset (mean ± standard deviation).

	Control			Mild amblyopia			Severe amblyopia		
	BE	LE	RE	BE	FE	AE	BE	FE	AE
Vector magnitude (mm)	82.64±27. 64	82.64±28.36	79.37±25.96	62.01±16.96	55.77±19.48	58.28±13.64	61.44±13.81	61.02±20.31	55.54±9.89
Vector variability (mm)	9.00±3.82	9.94±3.94	9.46±5.26	8.57±3.80	7.51±2.64	9.13±3.87	8.72±4.36	9.42±5.56	8.69±3.47
Angle magnitude (°)	1.29±0.04	1.28±0.04	1.28±0.04	1.24±0.04	1.22±0.05	1.23±0.04	1.25±0.04	1.24±0.05	1.23±0.04
Angle variability (°)	0.016±0.01	0.019±0.01	0.016±0.01	0.27±0.01	0.031±0.02	0.031±0.02	0.022±0.01	0.029±0.03	0.029±0.01

BE: binocular; LE: left eye; RE: right eye; FE: fellow eye; AE: amblyopic eye.

Mean *magnitude* of the reach vector was significantly different among Groups (F_(2,36)_ = 5.30, p = 0.009); the mean magnitude was significantly higher in control participants (81.09±27.28 mm), in comparison to patients with mild (58.68±17.01 mm) and severe (59.34±15.47 mm) amblyopia. There was no significant difference in the *variability* of the magnitude of the reach vector (F_(2,36)_ = 0.42, p = 0.657).

The *mean angle* between the reach vector and target vector was significantly different among Groups (F_(2,36)_ = 6.02, p = 0.006); the mean angle between the vectors was significantly higher in control participants (1.28±0.04°), compared to patients with mild (1.23±0.05°) and severe (1.24±0.04°) amblyopia. There was also a significant main effect among Groups for the *variability* of the vector angle (F_(2,36)_ = 7.15, p = 0.002; Figure 3b); the variability of the vector angle was significantly lower in control participants (0.018±0.01°), compared to patients with mild (0.030±0.02°) and severe (0.027±0.02°) amblyopia.

#### End of Movement

There was no significant difference in the *magnitude* of the reach vector at the end of movement (F_(2,36)_ = 2.43, p = 0.102). The *variability* of the magnitude of the reach vector (endpoint precision) was also not significantly different among Groups (F_(2,36)_ = 2.80, p = 0.074). The interaction between Group and Viewing Condition was also not significant (F_(4,72)_ = 1.99, p = 0.106).

**Figure 4:**
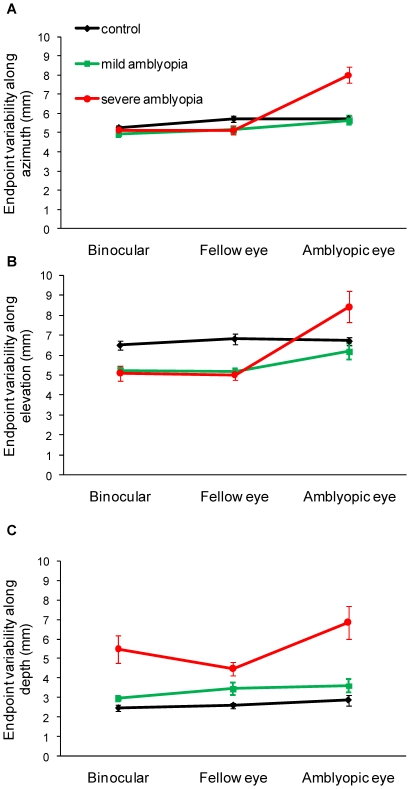
End-point precision. Mean endpoint precision (variable error) of the reaching movement along the azimuth (A), elevation (B), and depth axes (C). Patients with severe amblyopia had reduced precision during amblyopic eye viewing along azimuth (p<0.0001) and elevation axes (p<0.05), and during all viewing conditions along the depth axis (p<0.01). For control subjects, fellow eye is the right eye and amblyopic eye is the left eye. Error bars = ±1 standard error.

We also examined the endpoint precision of the 3 components of the vector (Table 5). A significant interaction between Group and Viewing Condition was found for *azimuth* (F_(4,72)_ = 8.85, p<0.0001; Figure 4a). Post-hoc tests indicated that control participants reached similar endpoint precision along the azimuth in all viewing conditions, which was not significantly different from patients with mild amblyopia. In contrast, patients with severe amblyopia had reduced precision when viewing with the amblyopic eye (7.61±3.01 mm), in comparison to binocular (4.91±1.90 mm) and fellow eye viewing (5.14±2.29 mm).

**Table 5:** Endpoint precision of the reach (mean ± standard deviation).

	Control			Mild amblyopia			Severe amblyopia		
	BE	LE	RE	BE	FE	AE	BE	FE	AE
Azimuth (mm)	5.23±1.49	5.72±1.74	5.68±1.92	4.95±1.64	5.15±1.85	5.66±2.43	4.91±1.90	5.14±2.29	7.61±3.01
Elevation (mm)	6.47±2.46	6.80±2.64	6.67±2.17	5.22±1.94	5.19±1.72	6.02±3.55	4.71±1.85	5.00±2.41	7.41±5.24
Depth (mm)	2.29±1.02	2.56±1.35	2.58±1.34	2.93±1.46	3.22±2.10	3.37±2.26	4.97±5.52	4.47±3.36	5.82±5.42

BE: binocular; LE: left eye; RE: right eye; FE: fellow eye; AE: amblyopic eye.

There was also a significant interaction between Group and Viewing Condition for *elevation* (F_(4,72)_ = 2.82, p = 0.031; Figure 4b). Post-hoc tests indicated control participants reached similar endpoint precision along elevation in all viewing conditions, which was comparable to patients with mild amblyopia. In contrast, patients with severe amblyopia had significantly reduced precision during amblyopic eye viewing (7.41±5.24 mm), in comparison to binocular viewing (4.71±1.85 mm) and fellow eye viewing (5.00±2.41 mm).

There was a significant main effect of Group for endpoint precision for *depth* (F_(2,36)_ = 7.70, p = 0.002; Figure 4c). Post-hoc tests indicated that patients with severe amblyopia had reduced precision in all viewing conditions (binocular: 4.97±5.52 mm; fellow eye: 4.47±3.36 mm; amblyopic eye: 5.82±5.42 mm), in comparison to control participants (binocular: 2.29±1.02 mm; left eye: 2.56±1.35 mm; right eye: 2.58±1.34 mm) and to patients with mild amblyopia (binocular: 2.93±1.46 mm; fellow eye: 3.22±2.10 mm; amblyopic eye: 3.37±2.26 mm).

### Correlation Analysis

#### Azimuth

**Figure 5:**
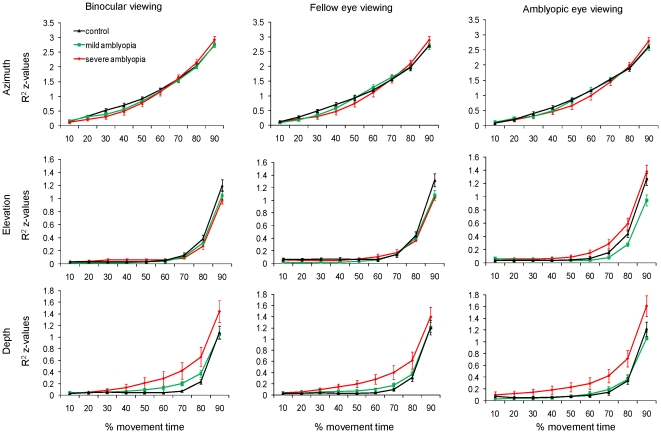
Proportion of explained variance. R^2^ values (Fisher z scores) relating the spatial location of the finger at 10% intervals (normalized to movement time) to the overall movement amplitude during binocular (left column), fellow eye (middle column) and amblyopic eye (right column) along the azimuth (top row), elevation (middle row), and depth axes (bottom row). There was no significant difference between control participants and patients with mild amblyopia in all viewing conditions in all three axes. However, patients with severe amblyopia had significantly higher R^2^ values at 70% of movement time along elevation (p<0.05) during amblyopic eye viewing, and along the depth axis (p<0.01) in all viewing conditions. The higher R^2^ values in the latter half of the trajectory indicate that movements relied heavily on pre-programmed responses.

Figure 5 (top row) shows the mean Fisher z scores (i.e., transformed R^2^ values) for control participants and patients from 10% to 90% of movement time. As depicted, the transformed R^2^ values increased towards the end of the movement; however, there were no significant differences among Groups in any viewing condition (F_(4,72)_ = 0.02, p = 0.982).

#### Elevation (Figure 5, middle row)

The interaction between Group and Viewing Eye was significant at 70% of movement time along elevation (F_(4,72)_ = 3.43, p = 0.013). Post-hoc tests showed that the proportion of endpoint variance explained at 70% of movement time (R^2^) was significantly higher for patients with severe amblyopia during amblyopic eye viewing (0.29±0.08), as compared to binocular (0.09±0.02) or fellow eye viewing (0.17±0.04). R^2^ values were similar across viewing conditions for control participants and patients with mild amblyopia.

#### Depth (Figure 5, bottom row)

There was a significant main effect of Group (F_(2,36)_ = 5.47, p = 0.008). Post-hoc tests revealed that patients with severe amblyopia had significantly higher R^2^ at 70% of movement time along the depth axis in all viewing conditions (binocular: 0.42±0.15, fellow eye: 0.40±0.12, amblyopic eye: 0.42±0.11), in comparison to control participants (binocular: 0.07±0.02, left monocular: 0.10±0.03, right monocular: 0.14±0.04) and to patients with mild amblyopia (binocular: 0.20±0.04, fellow eye: 0.17±0.05, amblyopic eye: 0.19±0.05). No difference was found between control participants and patients with mild amblyopia.

### Relationship between visual acuity and reach outcome measures

There was no significant relationship between acuity and vector angle variability early in the trajectory at 50 ms (r = 0.23, p = 0.265) or at 100 ms after movement onset (r = 0.09, p = 0.667). The relationship between acuity and online control (i.e., the transformed R^2^ values) was also not significant (azimuth: r = 0.17, p = 0.402; elevation: r = 0.17, p = 0.404; depth: r = 0.06, p = 0.773). However, there was a significant relationship between acuity and endpoint variability in depth (r = 0.51, p = 0.007).

A significant correlation was also found between acuity and reach reaction time (r = 0.42, p = 0.030), but not for the other reach outcome measures: movement time (r = −0.16, p = 0.430), peak acceleration (r = 0.16, p = 0.445), or duration of deceleration phase (r = −0.20, p = 0.319).

## Discussion

In this study, we investigated the effects of amblyopia on motor planning and online control during unconstrained, visually-guided reach-to-touch movements. The major findings are: (1) regardless of the severity of their visual deficits, patients showed reduced precision of the reach vector angle early in the trajectory in all viewing conditions; (2) patients with mild amblyopia attained normal endpoint reach precision in all viewing conditions; (3) patients with severe amblyopia had reduced endpoint precision along the azimuth and elevation axes during amblyopic eye viewing only, but they had reduced endpoint precision along the depth axis during all viewing conditions; (4) patients with severe amblyopia had significantly higher R^2^ values along the elevation and depth axes during amblyopic eye viewing, as compared to patients with mild amblyopia and control participants. Taken together, these data indicate that increased uncertainty in visual input due to amblyopia leads to reduced precision of the motor plan. In addition, patients with severe acuity deficits had reduced ability to use online control to modify their reach trajectory.

### Effect of amblyopia on motor planning

We previously reported that patients with anisometropic amblyopia modified the kinematics of their reaching movements by reducing peak acceleration and extending the duration of the acceleration phase when reaching to a high contrast visual target [57]. Here we extend our previous work by showing that positional uncertainty of the visual input due to amblyopia affects motor planning, as shown by increased variability of the reach vector angle early in the movement trajectory.

The effects of amblyopia on spatial perception have been examined using variety of tasks, including vertical alignment [58], [59], line bisection [60], [61], [62], [63], Vernier acuity [62], [64] and localization [65]. These studies reported a range of deficits in terms of distortions, as well as reduced accuracy and precision of spatial localization during amblyopic eye viewing. Given that target localization is the first stage in the generation of visuomotor behaviour, it is not surprising to find that our patients had significantly reduced precision of the early trajectory during *amblyopic eye* viewing.

A more surprising finding is that the initial trajectory also had reduced precision during *binocular and fellow eye* viewing. This is in contrast to most studies which found that detection of peripheral targets and perceptual localization were better during binocular and fellow eye viewing, in comparison to amblyopic eye viewing [58], [62], [65], [66]. The discrepancy between our results and those from previous studies is most likely due to the differences between tasks. Previous studies examined spatial localization using perceptual tasks that involved detection and/or discrimination of grating stimuli or Gaussian blobs, whereas participants in our study were required to make fast and accurate reaching movements to a single, high contrast target. The dissociation between visual pathways mediating spatial perception (i.e., ventral stream) and action (i.e., dorsal stream) is well established and supported by neuroanatomical studies [67] and behavioral data [68], [69]. Our findings show similar reduction in the precision of the initial trajectory in all viewing conditions suggesting that amblyopia might have greater detrimental effect on processing of information within the dorsal stream than the ventral stream during motor planning, independent of which eye is viewing.

In contrast to a few studies on spatial perception which reported greater performance decrements in patients with worse acuity [61], [70], we found no differences between patients with mild and severe amblyopia in terms of reduced precision during early reach trajectory. Also, consistent with the results in our previous study with a smaller sample size [57], we found no differences between patients with mild and severe amblyopia for other kinematic parameters which reflect the planning stage of the movement, including peak acceleration, peak velocity, and the duration of the acceleration phase. In fact, both groups of patients showed similar changes in the early kinematic parameters in comparison to age-matched control participants. The only difference that was found between patients with mild and severe amblyopia was for the reach reaction time, which was significantly longer for patients with severe amblyopia in all viewing conditions. The reach reaction time was correlated significantly with visual acuity—patients with worse acuity had longer reaction times. These results indicate that patients with worse acuity may have needed a longer time to detect the target and to plan the reach, but once the movement was initiated, all patients showed the same reach strategy characterized by longer movement time and lower peak acceleration.

Our results can be interpreted by considering recent studies which examined the interaction between sensory and motor uncertainty in healthy people. These studies have shown that participants are generally aware of the uncertainty of the visual cues [71], [72] and in their sensory modalities [73], [74], [75], which leads to task dependent optimal combination of sensory information [76]. In the motor system, a minimum variance model has been proposed which states that the variability in motor output scales with the magnitude of the command signal (i.e., signal-dependent noise) [77]. In addition, it has been shown that visually normal people can estimate and compensate for their movement variability [78]. Furthermore, visually normal people use a near-optimal trade-off strategy when combining sensory and motor uncertainty in tasks where the time allotted to perception and action is limited [79], [80]. Our study extends these findings by showing that the sensorimotor trade-off strategy is also evident in patients with amblyopia. The reduced precision of the motor plan in patients is most likely due to transformation of a degraded visual signal into a noisy motor plan, which in turn affects the precision of the motor command. Patients with mild amblyopia compensate for the increased uncertainty of the visual signal by reducing their motor output, allowing them to attain relatively good accuracy and precision at the end of the movement, regardless of viewing condition.

### Effect of amblyopia on online control

During movement execution, visual and proprioceptive feedback are integrated with predictions generated from the internal model, which are used to update the motor plan to correct for errors in the initial motor command and/or unexpected perturbations [14], [81], [82], [83]. The accuracy and precision of reaching movements ultimately depend on the ability to implement online control/corrections. In this study, we found that patients' ability to use online control to compensate for the reduced precision of the initial motor plan was dependent on the viewing condition, the severity of amblyopia, and the axis of the reach.

Patients with mild amblyopia were able to compensate for the early errors and achieved similar precision to control participants along the azimuth, elevation and depth axes in all viewing conditions. The correlation analysis (i.e., R^2^ values) showed no difference between control participants and patients with mild amblyopia, indicating that both groups used a comparable control strategy. In particular, the lower R^2^ values combined with good endpoint precision suggest that patients with mild amblyopia were able to effectively implement online corrections in the deceleration phase of the reaching movement, which allowed them to attain good endpoint precision and accuracy.

In contrast, patients with severe amblyopia had significantly reduced precision at the end of the movement along azimuth and elevation during amblyopic eye viewing. The loss of precision along the elevation axis together with significantly higher R^2^ values suggest that patients with severe amblyopia were not able to use visual feedback effectively during the movement to correct their trajectory. Surprisingly, despite increased endpoint variability along the azimuth, we found no difference in the R^2^ values among groups or viewing conditions. At least two explanations can account for these results. First, it is possible that patients adopted an online control strategy but they were not successful in implementing corrections because their visual feedback signal was severely degraded. Second, it is possible that the extent of the trajectory along the azimuth was too short (∼4 cm for the 5° target and ∼8 cm for the 10° target) to reveal differences in the control processes.

For patients with severe amblyopia, the increase in endpoint variability was particularly apparent along the depth axis in all viewing conditions, which is most likely related to the fact that they have abnormal stereopsis (11 of 13 patients had no clinically detectable stereopsis, while the other two had gross stereopsis of 3000 arc sec only). These results are consistent with previous studies which found a relationship between poor stereoacuity and performance degradation on clinical tests of motor skills [46], [84]. In addition, previous studies reported that patients with reduced stereopsis exhibit deficits when executing 3D grasping movements [49], [50], [85]. Specifically, Suttle and colleagues [50] reported that children with amblyopia made more errors when reaching and grasping for an object and consequently collided with the objects more frequently. These errors were evident in all viewing conditions and were most pronounced in children with poor binocular vision. The authors suggested that children with stereoacuity deficits were not able to use dynamic binocular cues to modify movement trajectory. Our study extends these findings by showing that these movement errors were most likely due to reduced ability to engage in online control, specifically along the depth axis.

The results from our study emphasize the importance of binocular vision for the online control of reaching movements. These results are consistent with other studies which found that binocular vision plays an important role in reaching movements [86], [87], [88], [89], [90], [91], [92], [93], [94], [95], [96]. Binocular disparity can be combined with vergence to estimate the distances of objects in depth when they are within the reaching space [97]. Relative disparity between the target and hand can also be used to guide the adjustments of the hand during the approach phase of a reaching movement [93], although the role of relative binocular disparity for fast online control of reaching has been questioned [95]. Nonetheless, a study that used a perturbation of a 3D surface during an object placement task found that participants initiated online corrections quicker based on binocular cues as compared to monocular cues [98]. The importance of binocular vision for online corrections was also confirmed in another study where participants executed 3D reaching movements while the target was perturbed in depth [91]. They showed that participants were able to initiate a corrective movement 230 ms after the perturbation during binocular viewing, but were not able to initiate corrections at all when only monocular cues were available.

In conclusion, the disruption of concordant binocular visual input during early development often results in visual impairment referred to as amblyopia. The consequences of spatiotemporal visual deficits in amblyopia can be readily observed in the performance of functional motor behaviours, such as reaching and grasping, walking and driving [99]. To our best knowledge, our study is the first to show explicitly that people with amblyopia exhibit a deficit in motor planning, most likely as a result of visual spatiotemporal uncertainty. Patients use a sensorimotor trade-off strategy to compensate for the increased spatiotemporal uncertainty in the visual signal. However, the degree of compensation depends on the severity of the deficit and the axis of the reaching movement, indicating that stereopsis plays a critical role. Results from this study provide insight into the sensorimotor control strategy for reaching movements and may have implications for the development of appropriate assessment and treatment protocols for amblyopia.
